# A Novel Gelatin Hydrogel‐Based Drug‐Delivery System Promotes Antimicrobial Efficacy in a Pyogenic Spondylodiscitis Rat Model

**DOI:** 10.1002/jsp2.70213

**Published:** 2026-09-01

**Authors:** Kunihiko Miyazaki, Yutaro Kanda, Takashi Yurube, Yoshiki Takeoka, Takeru Tsujimoto, Hiroki Ohnishi, Tomoya Matsuo, Masao Ryu, Naotoshi Kumagai, Kohei Kuroshima, Yoshiaki Hiranaka, Yasuhiko Tabata, Ryosuke Kuroda, Kenichiro Kakutani

**Affiliations:** ^1^ Department of Orthopaedic Surgery Kobe University Graduate School of Medicine Kobe Japan; ^2^ Laboratory of Biomaterials, Department of Regeneration Science and Engineering, Institute for Life and Medical Sciences Kyoto University Kyoto Japan

**Keywords:** antibiotics, drug delivery system, gelatin hydrogel, spondylitis, spondylodiscitis

## Abstract

**Background:**

Spondylodiscitis/spondylitis is an intractable disease requiring long‐term intravenous antibiotics administration. However, drug delivery is greatly restricted because of the avascular nature in the intervertebral disc, which not only delays recovery but increases antimicrobial resistance and osteomyelitis. The purpose of this study is to elucidate the efficacy of gelatin hydrogel microsphere incorporating cefazolin (GM‐CEZ) using a rat spondylodiscitis model.

**Methods:**

First, GM‐CEZ was characterized by in vitro degradability, sustainability, and antimicrobial effects. Second, its efficacy in vivo was evaluated using a rat spondylodiscitis model of bioluminescent methicillin‐susceptible *Staphylococcus aureus* . Antimicrobial effects were monitored using an in vivo imaging system and blood tests. Residual bone volume was evaluated using micro‐computed tomography. Immunohistology for angiogenesis‐related CD31 and smooth muscle actin was also performed. Third, in vivo safety was evaluated using a rat intervertebral disc injection model with blood tests for systemic safety and TUNEL staining for local safety.

**Results:**

In vitro, CEZ was gradually released with GM degradation and antimicrobial effects increased proportionally to the degradation. In vivo, single topical injection of GM‐CEZ (1 mg GM, 250 μg/kg CEZ, 100 μL collagen) significantly reduced the amount of bacteria with fewer white blood cells 7 days after treatment and bone destruction 28 days after treatment, compared to local administration of CEZ (250 μg/kg) and systemic administration of CEZ (25 mg/kg/day for 28 days) (all, *p* < 0.05). Immunopositivity was significantly higher in rats with local GM‐CEZ administration than those with local CEZ and systemic CEZ administration (all, *p* < 0.05), indicating greater tissue reformation. In vivo safety experiments demonstrated no significant myelotoxicity, nephrotoxicity, and local cytotoxicity of GM‐CEZ.

**Conclusions:**

The local administration of GM‐CEZ using a fraction of the systemic CEZ dose achieved enhanced antimicrobial effects without systemic and local toxicity, suggesting its potential as a promising treatment strategy for pyogenic spondylodiscitis.

## 1 Introduction

Spondylodiscitis is the most common manifestation of spinal infection [1], with an incidence rate of 0.2–4.4 cases per 100 000 per annum and a high mortality rate of up to 20% [2, 3, 4].

Several recent studies reported an alarming increase of incidence in the last 20 years [5]. Pyogenic spondylodiscitis is the most common subtype and most commonly caused by *Staphylococcus aureus* , followed by *Escherichia coli* [6]. Pyogenic spondylodiscitis affects the intervertebral disc (IVD) space and nearby vertebrae, leading to osteomyelitis and surrounding soft tissue infection including abscess in epidural space and iliopsoas when early healing fails. Because prolonged infection also leads to bone destruction with spinal instability and compression of neurological structures, timely diagnosis of the causative bacteria and appropriate intervention are essential. Long‐term antibiotics administration for at least 6 weeks with long‐term bed rest is the mainstay of treatment [4]. Because the most common causative bacteria are methicillin‐susceptible *Staphylococcus aureus* (MSSA), cefazolin (CEZ) is the gold‐standard antibiotic in the treatment of pyogenic spondylodiscitis [6]. Surgeries are sometimes performed for direct debulking of bacteria and local rest with spinal stabilization. However, long‐term high‐dose antibiotics administration and bed rest hamper patients' quality of life, cause social and economic losses [7], and may increase the risk of systemic adverse effects such as nephrotoxicity [8]. Therefore, it is highly desirable to find a new therapy to shorten the treatment duration for pyogenic spondylodiscitis.

Osteomyelitis and deep soft tissue infection are difficult‐to‐treat infectious diseases. Considering the side effects and drug resistance of systemic antibiotics, local drug delivery systems—sustained release of drugs at local sites for enhancing drug effects with minimizing harmful side effects associated with systemic administration—have been explored [9, 10]. Furthermore, as the IVD is the largest avascular organ in the whole body [11], the efficacy of systemic antibiotic administration to the infection site is greatly restricted. Therefore, a drug delivery system can be a most powerful tool to treat spondylodiscitis. However, little evidence regarding a drug delivery system for treating pyogenic spondylodiscitis exists. We previously demonstrated the efficacy of gelatin hydrogel microspheres (GM) as a drug delivery system for local sustained release of anticancer drugs to target metastatic tumors [12]. In this study, we aimed to elucidate the in vitro characteristics of GM incorporating CEZ (GM‐CEZ) and its in vivo efficacy using a rat MSSA spondylodiscitis model.

## 2 Materials and Methods

### 2.1 Ethics Statement

All animal experiments were performed under the approval and guidance of the Institutional Animal Care and Use Committee at Kobe University Graduate School of Medicine (Kobe, Japan) (approval no. P190708‐R1).

### 2.2 Bacteria

We selected MSSA as a bacterial strain, which is the most common [6] causative pathogen of pyogenic spondylodiscitis. Luminescent XEN29 *Staphylococcus aureus* was purchased (ATCC 49525; Perkin Elmer, Waltham, MA, USA) to use in vitro and in vivo experiments. Before inoculation, the bacterial colony was suspended in 5 mL of LB medium (LB Broth, Sigma‐Aldrich, St. Louis, MO, USA) and cultured with shaking at 120 rpm in a 37°C water bath for 20 h. After incubation, 5 mL of 30% glycerol solution was added to the culture and thoroughly mixed. A bacterial solution in phosphate‐buffered saline (PBS) (1.0 × 10^6^ Colony Forming Unit [CFU]/0.1 mL) was prepared and stored on ice until administration [13].

### 2.3 Animals

**FIGURE 1:**
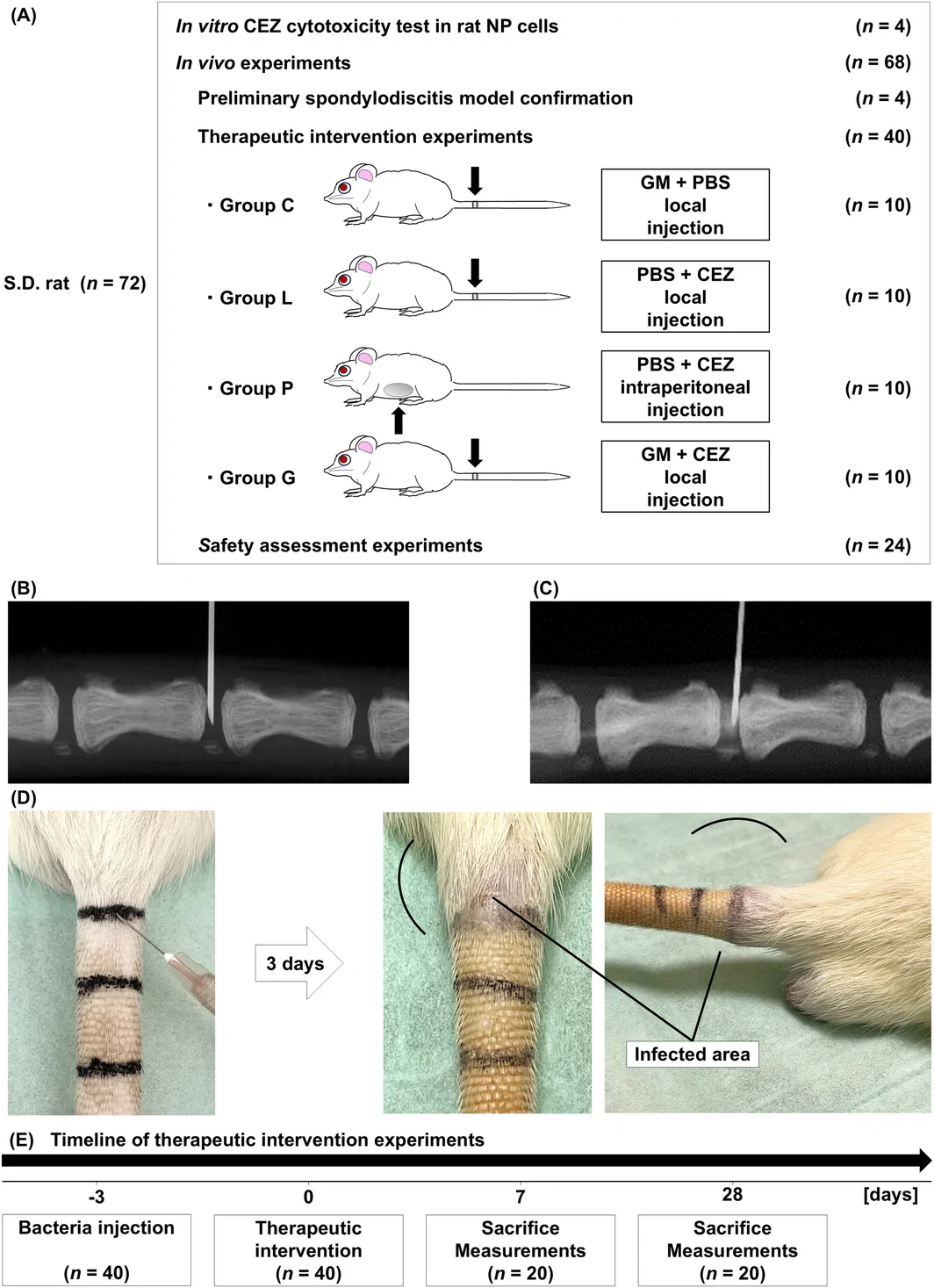
(A) Schematic overview of experimental workflow. S.D. rats were used (*n* = 72) as follows: In vitro CEZ cytotoxicity test in rat NP cells (*n* = 4), preliminary model confirmation (*n* = 4), therapeutic intervention experiments using the rat spondylodiscitis model (*n* = 40), and safety assessment experiments (*n* = 24). In the therapeutic intervention experiments, 40 rats were randomly assigned to the following four groups (*n* = 10 per group): Group C, local administration of GM‐PBS; group L, local administration of CEZ solution (250 μg/kg, calculated for CEZ); group P, intraperitoneal injection of CEZ solution (25 mg/kg/day, calculated for CEZ) for 28 days; group G, local administration of GM‐CEZ (250 μg/kg, calculated for CEZ). S.D., Sprague–Dawley; CEZ, cefazolin; NP, nucleus pulposus; GM, gelatin hydrogel microspheres; PBS, phosphate‐buffered saline. (B), (C) Plain radiographs before (B) and after (C) contrast agent injection into the center of intervertebral disc using a 27G sterile needle in a rat. (D) Photographs of a rat tail before and after bacteria injection into the C8/9 disc. Three days after injection, soft tissue around the infected intervertebral disc was swollen. (E) The timeline of therapeutic intervention experiments.

Seventy‐two twelve‐week‐old male Sprague–Dawley rats purchased from Japan SLC Inc. (Shizuoka, Japan) were used. Four rats were used for in vitro experiments, and sixty‐eight rats were used for in vivo experiments, including preliminary model confirmation (*n* = 4), therapeutic intervention experiments (*n* = 40), and safety assessment experiments (*n* = 24) (Figure 1A). Rats were housed in an air‐conditioned room with a 12‐h light/dark cycle. Animals were fed pathogen‐free laboratory chow and had free access to autoclaved water.

### 2.4 Cells and In Vitro Cytotoxicity of CEZ

Rat IVD nucleus pulposus (NP) tissue was harvested from rat coccygeal IVD (*n* = 4) after euthanasia and digested in 1% penicillin–streptomycin (26253–84; Nacalai Tesque, Kyoto, Japan) supplemented Dulbecco's modified Eagle's medium (DMEM; D5796; Sigma‐Aldrich, St. Louis, MO, USA) with 10% fetal bovine serum (FBS; F2442; Sigma‐Aldrich) and 0.114% collagenase type II (LS004176; Worthington Biochemical, Lakewood, NJ, USA) for 1 h at 37°C [14]. Isolated NP cells were cultured as a monolayer on DMEM supplemented with 1% penicillin–streptomycin with 10% FBS at 37°C under 2% O_2_ to stimulate a physiologically hypoxic IVD environment until the cells reached 80% confluence. To retain the phenotype, only first‐passage cells were used for evaluation.

To determine whether the highest concentration could maintain antimicrobial activity of CEZ without exhibiting cytotoxicity, the in vitro viability of IVD NP cells at different CEZ concentrations was assessed. NP cells were seeded on a 96‐well plate at the density of 5.0 × 10 [3]/well. After 72 h pre‐culture, NP cells were treated with various concentrations of CEZ up to 1.0 × 10^4^ μg/mL for 24 h. Cell viability was then assessed using a Cell Counting Kit‐8 (Dojindo, Kumamoto, Japan). Experiments were performed with four replicate wells and measurements were conducted three times for each well.

### 2.5 Preparation of GM‐CEZ

GMs were prepared as described previously [13]. Briefly, gelatin (beMatrix LS‐H; Nitta Gelatin Inc., Osaka, Japan) solution (5 wt%) in double deionized water (DDW) was cast into Tissue‐Tek mold (Sakura Finetek Japan Co. Ltd., Tokyo, Japan) and then freeze dried (Takara, Tokyo, Japan) for 72 h. Dehydrothermal crosslinking (Aurora DN‐30S, Sato vac Inc., Tokyo, Japan) was performed at 140°C for 48 h instead of chemical crosslinking to eliminate the possibility of its toxicity in the body. The crosslinked gelatin hydrogels were homogenized using a Polytron PT‐13000DM system (Central Scientific Commerce, Tokyo, Japan) and separated by sieves to obtain an injectable GM (100–180 μm) in a DDW‐swollen state. The resulting GM was further freeze‐dried for 48 h, followed by ethylene oxide sterilization. To prepare GM incorporating CEZ (GM‐CEZ), the cross‐linked GM (1 mg) was immersed in 10 μL CEZ solution (1.0 × 10^4^ μg/mL) for 1 h at 37°C and freeze‐dried for another 48 h. Freeze‐dried GM‐CEZ was directly mixed with 100 μL collagen solution at 4°C to reduce leakage at the injection site immediately before use for both in vitro and in vivo experiments. This composite remained sufficiently fluid at 4°C and was successfully injected through a 27‐gauge needle without apparent needle obstruction or difficulty in extrusion as well as freeze‐dried GM‐CEZ mixed with PBS.

### 2.6 Characterization of GM‐CEZ In Vitro

The characterization of GM‐CEZ was performed by in vitro degradability and release tests. First, GM‐CEZ (GM 1 mg, CEZ 1.0 × 10^2^ μg, collagen 100 μL) was placed in 1 mL of 0.01 mol/L phosphate‐buffered saline (PBS) containing 0.01% Tween 80, followed by reciprocal shaking at 60 strokes/min at 37°C. At each time point (1, 2, 24, 25, 26, 30, 36, 48, 60, 72, 84 h), the supernatant was collected and replaced with the same volume of PBS. After 24 h, PBS was replaced with PBS containing collagenase D (25 μg/mL). The degradability of GM‐CEZ was assessed by quantitating proteins in the supernatant with a bicinchoninic acid protein assay kit (Pierce BCA Protein Assay Kits; A55864). The concentration of released CEZ at each time point was measured using high‐performance liquid chromatography [15]. Experiments were performed using four replicate wells and measurements were conducted three times for each well. The antimicrobial effect of CEZ released from GM‐CEZ against MSSA was evaluated by measuring the zone of inhibition on agar plates using the disc diffusion test [16]. As described above, GM‐CEZ (GM 1 mg, CEZ 1.0 × 10^2^ μg, collagen 100 μL) was placed in 1 mL of 0.01 mol/L PBS containing 0.01% Tween 80, followed by reciprocal shaking at 60 strokes/min at 37°C. According to the results of in vitro degradability and release tests, the supernatant was collected at 2, 26, and 84 h without replacement of PBS to prepare released CEZ from GM‐CEZ samples at various stages of degradation (2, initially released; 26 h: partially released; 84 h: totally released). Collagenase D (25 μg/mL) was administered at 24 h. 100 μL luminescent XEN29 *Staphylococcus aureus* suspension (1.0 × 10^6^ CFU/mL) was applied to a plate of preheated nutrient agar medium (Unitech, MA, USA) at 37°C. A paper disc soaked in the supernatant containing CEZ was placed on the agar plate before incubation at 37°C. After 24 h, the zone of inhibition was measured using a slide caliper. Measurements were conducted two times for each zone. Four identical experiments were conducted.

### 2.7 Rat Pyogenic Spondylitis Model

We used an established rat pyogenic spondylitis model [13]. XEN‐29 *Staphylococcus aureus* suspension (1.0 × 10^6^ CFU in 100 μL PBS) prepared in vitro was carefully injected into the center of IVD at the C8–C9 level in rats under general anesthesia using medetomidine, midazolam and butorphanol at a dose of 0.3 mg/kg + 4.0 mg/kg + 5.0 mg/kg (body weight) with isoflurane using a 27‐G sterile needle [13, 16]. Preliminary, four rats were used to confirm the accuracy of the intradiscal injection of bacteria with a contrast agent (Figure 1B,C). Forty rats (body weight: 349.6 ± 8.0 [333–367] g) were used to compare the effects of the therapeutic intervention. Three days after the bacteria injection, swelling was shown at the injected disc level (Figure 1D).

### 2.8 In Vivo Therapeutic Intervention Experiments

Three days after inoculation, rats were randomly assigned to the following four groups (*n* = 10 per each group): group C, local administration of GM‐PBS (GM, 1 mg; PBS, 10 μL; collagen 100 μL); group L, single topical injection of 100 μL CEZ solution (250 μg/kg, calculated for CEZ); group P, intraperitoneal injection of 5.0 mL CEZ solution (25 mg/kg/day, calculated for CEZ); group G, single topical injection of GM‐CEZ (GM, 1 mg; 250 μg/kg, calculated for CEZ, collagen 100 μL). The concentration of CEZ was determined based on in vitro cytotoxicity experiment and body weight. In the local administration groups (Groups L and G), a slightly lower concentration of 9.0 × 10^2^ μg/mL compared to the maximum concentration without in vitro cytotoxicity of 1.0 × 10^3^ μg/mL was selected to provide a safety margin. The concentration of 9.0 × 10^2^ μg/mL in a 100‐μL injection solution corresponded to 250 μg/kg. The dose of CEZ for systemic administration (group P) was determined based on the clinical dose (20–40 mg/kg, calculated for CEZ). Therapeutic intervention was performed 3 days after bacteria inoculation, because the amount of bacteria typically reached the maximum load 3 days after injection [13]. Injections into the IVD were performed with a 27G needle in the same way as the bacteria injection, and intraperitoneal injections were performed twice a day for 28 days with a 27G needle (Figure 1D). The timeline of therapeutic intervention experiments was shown in Figure 1E.

### 2.9 In Vivo Antimicrobial Effects

Rats in each group were euthanized at 7 or 28 days after treatment with an overdose of intraperitoneal injection of 1 mL pentobarbital sodium (*n* = 5 per each group). To assess the volume of XEN‐29 *Staphylococcus aureus* bacteria, fluorescent signal intensity was measured using an In vivo imaging system (IVIS) (Lumina II, Perkin Elmer) [12]. The total radiant efficiency (photons/s/mW) of the infected area was measured as the volume of XEN‐29 *Staphylococcus aureus* bacteria using living image software. Considering that pyogenic spondylodiscitis leads to osteomyelitis and osteolysis, residual bone volume of the spinal segment consisting of the affected intervertebral disc and its two adjacent vertebrae (one superior and one inferior) was quantitatively assessed using a micro‐computed tomography [μCT] scanner (R_mCT2; Rigaku, Tokyo, Japan). The bone volume fraction (BV/TV), a representative index in μCT analysis, was calculated using an image analysis system (TRI/3D‐BON; Ratoc Systems Engineering, Tokyo, Japan).

### 2.10 Immunohistology

The rat functional spinal units (vertebra‐IVD‐vertebra) were resected, fixed in 4% paraformaldehyde for 1 day, decalcified in 10% ethylenediaminetetraacetic acid (163‐20145; Wako) for 14 days, embedded in paraffin, and 7 μm mid‐sagittal sections were prepared for immunohistochemistry. Multicolor immunofluorescence was performed to assess vascular growth, which is essential for tissue reformation. After deparaffinization and heat‐induced epitope retrieval with Dako Target Retrieval Solution (Wako) at 90°C for 10 min, sections were incubated with 3% hydrogen peroxide and then incubated with 1:200 diluted primary antibodies against CD31 (bs‐0195R, Bioss Inc., MA, USA) and smooth muscle actin (SMA) (BF9212, Affinity Biosciences, OH, USA) for 12 h at 4°C. Then, sections were treated with Alexa Fluor 488‐conjugated and Alexa Fluor 594‐conjugated secondary antibodies for 1 h at room temperature. 4′,6‐diamidino‐2‐phenylindole (DAPI) (D1306; Thermo Fisher Scientific, MA, USA) was used for counterstaining. Images were obtained using a BZ‐X700 microscope (Keyence, Osaka, Japan). Using BZ‐X700 software, the number of positive cells was counted in four random high‐power fields (×400). Immunopositivity was calculated as the percentage relative to DAPI‐positive cells (*n* = 5 for each group).

### 2.11 Systemic Assessment in the Therapeutic Experiments

The white blood cell (WBC) count was measured to evaluate the degree of systemic inflammation on the day of *Staphylococcus aureus* administration, at the time of treatment, and on Days 7 and 28 (*n* = 5 per each group). The rats were weighed to assess general condition and systemic side effects at the same time points as blood tests (*n* = 5 per each group).

### 2.12 In Vivo Safety Experiments

To assess in vivo systemic and local safety after local administration into the IVD, rats were assigned to four groups (*n* = 6 per each group): PBS‐only group (PBS, 100 μL), GM‐PBS group (GM, 1 mg; PBS, 10 μL; collagen, 100 μL), CEZ group (100 μL CEZ solution, 250 μg/kg, calculated for CEZ), and GM‐CEZ group (GM, 1 mg; CEZ, 250 μg/kg; collagen, 100 μL). Injections into the IVD were also performed with a 27G needle. Rats in each group were euthanized at 3 or 7 days after local administration with an overdose of intraperitoneal injection of 1 mL pentobarbital sodium (*n* = 3 per each group).

For systemic safety assessment, peripheral blood samples were collected 3 and 7 days after local administration. Hematological parameters, including WBC, red blood cell (RBC), hemoglobin concentration, and platelet count, were measured to evaluate myelotoxicity. Serum biochemical parameters, including blood urea nitrogen (BUN) and creatinine, were measured to evaluate nephrotoxicity.

For local safety assessment, the rat functional spinal units were also resected after euthanasia, fixed in 4% paraformaldehyde for 1 day, decalcified in 10% ethylenediaminetetraacetic acid (163‐20145; Wako) for 14 days, embedded in paraffin, and 7 μm mid‐sagittal sections were prepared for terminal deoxynucleotidyl transferase dUTP nick‐end labeling (TUNEL) staining to detect cell apoptosis. TUNEL staining was performed with the Alexa Fluor 594‐labeled TUNEL assay kit (C10618; Thermo Fisher Scientific) according to the manufacturer's protocol, with DAPI used for nuclear counterstaining. TUNEL‐positive and DAPI‐positive cells were quantified in four random high‐power fields (×400), and the percentage of TUNEL‐positive cells was calculated.

### 2.13 Statistical Analysis

Data are expressed as the mean ± standard deviation (SD). For in vitro cytotoxicity test, in vitro antimicrobial analysis, and in vivo analyses, including IVIS and μCT, immunopositivity, TUNEL positivity, and blood tests, one‐way analysis of variance (ANOVA) and the Tukey–Kramer post hoc test were performed to compare differences among the groups. For the body weight, the Kruskal–Wallis test was used to compare differences among the four groups at each time point. A *p*‐value < 0.05 was considered statistically significant for all tests. Statistical analyses were conducted using SPSS version 29.0 software (IBM Corp., Armonk, NY).

## 3 Results

### 3.1 In Vitro Cytotoxicity of CEZ

**FIGURE 2:**
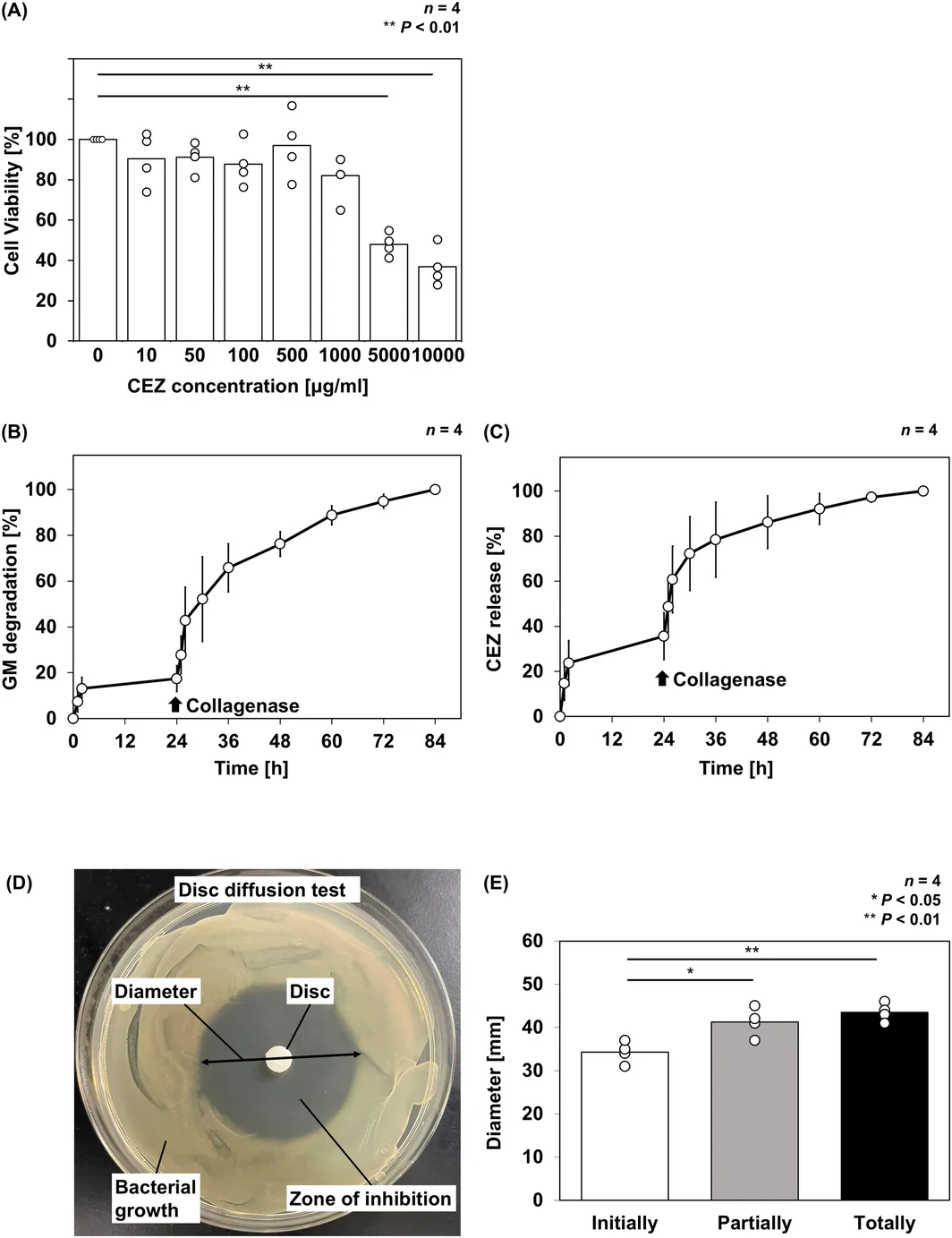
(A) In vitro CEZ cytotoxicity test in rat NP cells using a Cell Counting Kit‐8 assay after 24 h of treatment with CEZ at concentrations ranging from 0 to 1.0 × 10^4^ μg/mL. Data are presented as mean ± SD. **p < 0.01; significant difference; one‐way ANOVA with Tukey–Kramer post hoc test (*n* = 4). (B) Time profiles of the degradation of GM incorporating CEZ. An arrow indicates the time point (24 h) at which collagenase was added. (C) Time profiles of released CEZ from GM incorporating CEZ. An arrow indicates the time point (24 h) at which collagenase was added. (D) A photograph of the disc diffusion test showing the zone of inhibition around the CEZ‐soaked paper for bacterial growth. (E) The diameter of the zone of inhibition around the soaked paper with initially released (2 h), partially released (26 h), and completely released CEZ (84 h). Data are presented as mean ± SD. *p < 0.05, **p < 0.01, significant difference; one‐way ANOVA with Tukey–Kramer post hoc test (*n* = 4). ANOVA, analysis of variance; CEZ, cefazolin; GM, gelatin hydrogel microspheres; NP, nucleus pulposus; SD, standard deviation.

In the cytotoxicity assessment using the CCK‐8 assay, 1.0 × 10^3^ μg/mL CEZ showed no visible cytotoxicity compared to the control in rat NP cells (*p* = 0.278), whereas 5.0 × 10^3^ μg/mL (47.9% ± 4.9%) and 1.0 × 10^4^ μg/mL (36.9% ± 8.4%) CEZ demonstrated significant decreases in cell viability compared to the control (*p* < 0.001) (Figure 2A).

### 3.2 In Vitro Characterization of GM‐CEZ

In the degradability test, GM started degrading immediately in PBS, and the rate of degradation reached approximately 20% at 24 h. After treatment with PBS containing collagenase, GM‐CEZ degraded rapidly and the rate of gelatin degradability at 84 h was 100% (Figure 2B). In the release test, initial bursts were observed at 23.7% ± 10.0% within the first 2 h. After adding collagenase, CEZ was rapidly released along with GM degradation. Finally, almost all CEZ was released at 84 h (Figure 2C).

### 3.3 In Vitro Antimicrobial Effects of GM‐CEZ

A zone of inhibition was observed around the disc impregnated with the supernatant at various stages of degradation, demonstrating the in vitro antimicrobial effects of CEZ released from GM‐CEZ (Figure 2D). The diameter around the initially released CEZ (34.2 ± 2.2 mm) was significantly smaller than that around the partially released CEZ (41.3 ± 2.9 mm) (*p* = 0.012). Additionally, the diameter around the totally released CEZ was further larger (43.5 ± 1.8 mm) (*p* < 0.01, compared to initially released CEZ) (Figure 2E). These findings indicate that the antimicrobial effects of CEZ were maintained after the embedding process by GM and increased proportionally to the degradation of GM‐CEZ.

### 3.4 In Vivo Assessment of the Antimicrobial Effects in Rat IVDs

**FIGURE 3:**
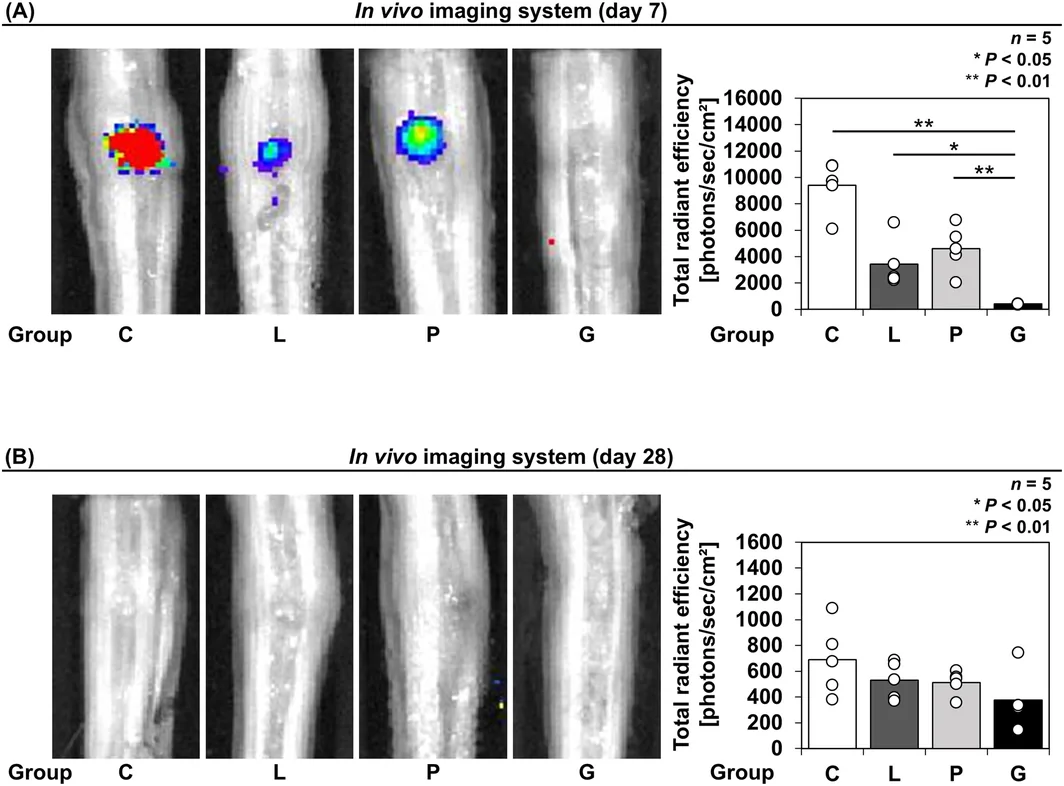
Bioluminescent intensity maps and quantified intensity captured using an IVIS imaging system at 7 (A) and 28 (B) days after four different treatments: Group C, local administration of GM‐PBS; group L, local administration of CEZ solution (250 μg/kg, calculated for CEZ); group P, intraperitoneal injection of CEZ solution (25 mg/kg/day, calculated for CEZ) for 28 days; group G, local administration of GM‐CEZ (250 μg/kg, calculated for CEZ). Bars indicate mean values, and circles indicate individual data points. *p < 0.05, **p < 0.01; significant difference; one‐way ANOVA with Tukey–Kramer post hoc test (*n* = 5). ANOVA, analysis of variance; CEZ, cefazolin; GM, gelatin hydrogel microspheres; IVIS, in vivo imaging system; PBS, phosphate‐buffered saline.

The infection sites in the four groups were detected using IVIS 7 days after treatment (Figure 3A). On Day 7 after treatment, all four groups showed a decrease in the number of bacteria. However, group C, which was not treated with antimicrobial agents, showed a significantly higher amount of bacteria compared to the other three groups (group C: 9404 ± 1759 photons/s/cm^2^; group L: 3418 ± 1648 photons/s/cm^2^, *p* < 0.01; group P: 4612 ± 1742 photons/s/cm^2^, *p* < 0.01; group G: 336 ± 34 photons/s/cm^2^, *p* < 0.01). Group G showed a significant decrease in the number of bacteria compared to groups P and L (P: *p* = 0.039; L, *p* < 0.01) (Figure 3A). On the other hand, there was no significant difference between the four groups on Day 28 (Figure 3B).

### 3.5 Assessment of Osteolytic Lesions

**FIGURE 4:**
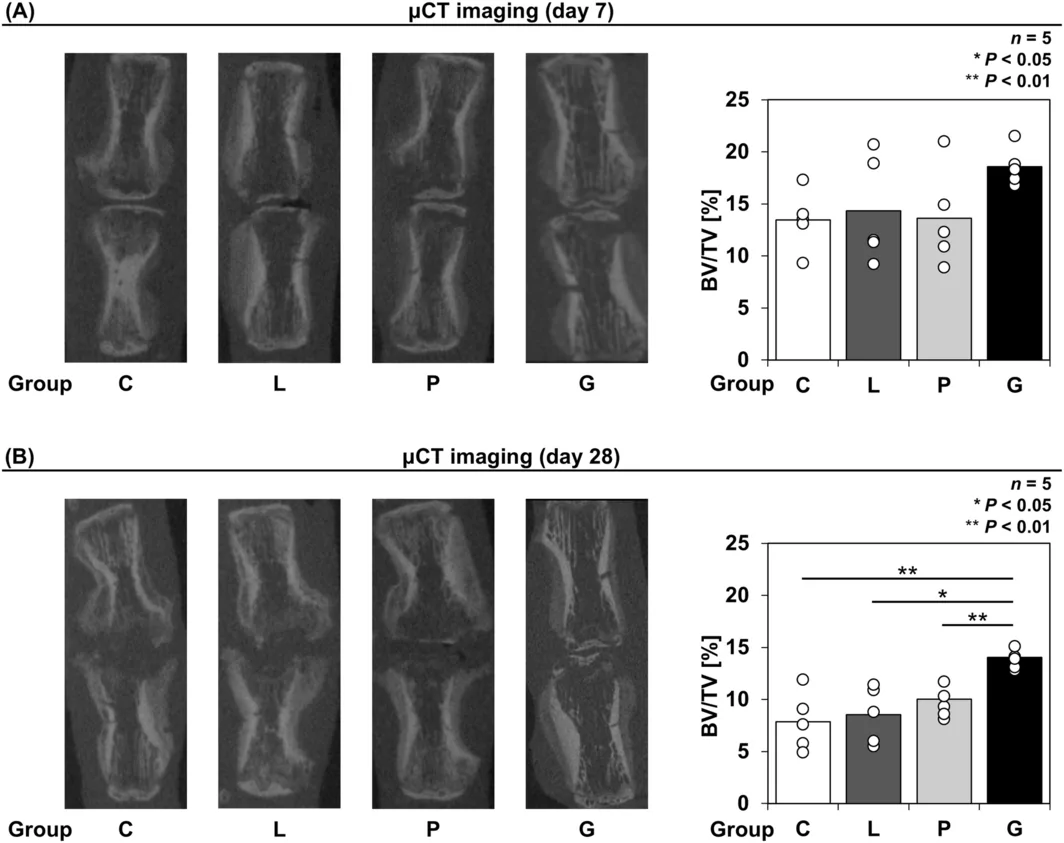
Bone morphometric images and quantified BV/TV (%) using a μCT scanner at 7 (A) and 28 (B) days after four different treatments: Group C, local administration of GM‐PBS; group L, local administration of CEZ solution (250 μg/kg, calculated for CEZ); group P, intraperitoneal injection of CEZ solution (25 mg/kg/day, calculated for CEZ) for 28 days; group G, local administration of GM‐CEZ (250 μg/kg, calculated for CEZ). Bars indicate mean values, and circles indicate individual data points. *p < 0.05, **p < 0.01; significant difference; one‐way ANOVA with Tukey–Kramer post hoc test (*n* = 5). ANOVA, analysis of variance; CEZ, cefazolin; CT, computed tomography; GM, gelatin hydrogel microspheres; PBS, phosphate‐buffered saline.

μCT analysis revealed no significant difference in BV/TV among the four groups on Day 7 after treatment (group C: 13.8% ± 4.3%; group L: 15.0% ± 3.5%; group P: 14.0% ± 4.6%; group G: 19.6% ± 3.3%) (Figure 4A). On Day 28, the mean BV/TV of group G was significantly higher than that of the other three groups (group C: 6.3% ± 2.1%, *p* < 0.01; group L: 8.4% ± 2.5%, *p* < 0.01; group P: 9.5% ± 1.2%, *p* = 0.02; group G: 13.5% ± 1.2%) (Figure 4B).

### 3.6 Immunofluorescent Staining

**FIGURE 5:**
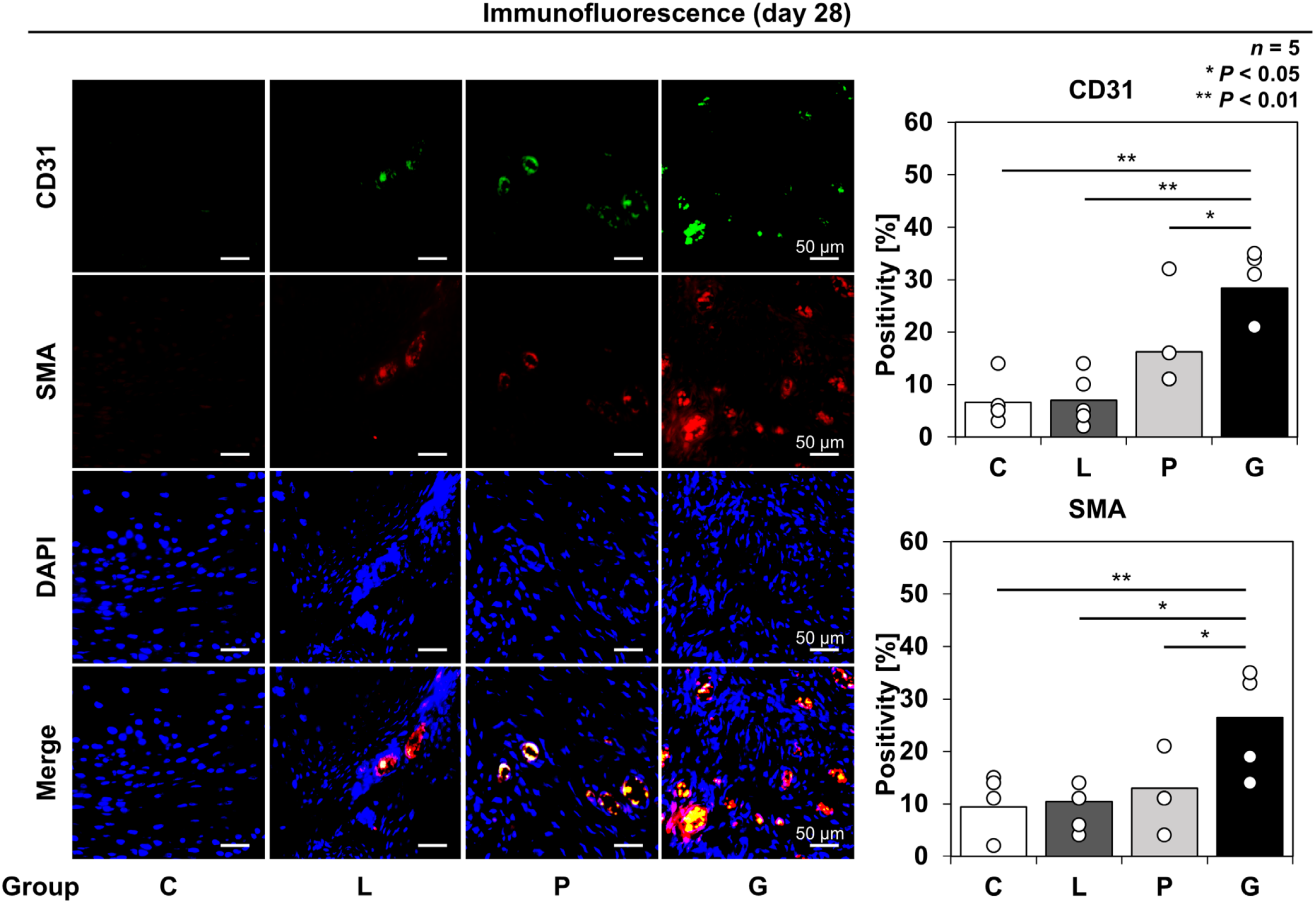
Immunofluorescence and immunopositivity of rat IVD at 28 days after four different treatments: Group C, local administration of GM‐PBS; group L, local administration of CEZ solution (250 μg/kg, calculated for CEZ); group P, intraperitoneal injection of CEZ solution (25 mg/kg/day, calculated for CEZ) for 28 days; group G, local administration of GM‐CEZ (250 μg/kg, calculated for CEZ) for CD31 (green), SMA (red), nuclear DAPI (blue), and merged signals. Bars indicate mean values, and circles indicate individual data points. *p < 0.05, **p < 0.01; significant difference; one‐way ANOVA with Tukey–Kramer post hoc test (*n* = 5). Scale bar, 50 μm. ANOVA, analysis of variance; CEZ, cefazolin; DAPI, 4′,6‐diamidino‐2‐phenylindole; GM, gelatin hydrogel microspheres; PBS, phosphate‐buffered saline; SMA, smooth muscle actin.

Tissue samples collected after 28 days were analyzed by immunofluorescence staining (Figure 5). The expression of the endothelial cell marker CD31 was markedly increased in group G (26.4% ± 8.3%) compared to the other three groups (group C: 9.4% ± 5.1%, *p* = 0.007; group L: 10.4% ± 4.8%, *p* = 0.011; group P: 13.0% ± 6.0%, *p* = 0.035). Additionally, immunopositivity of another endothelial cell marker SMA in group G (28.4% ± 6.2%) was also significantly higher than that in the other three groups (group C: 6.6% ± 3.8%, *p* < 0.001; group L: 7.0% ± 4.4%, *p* = 0.001; group P: 16.2% ± 8.1%, *p* = 0.043) (Figure 5). These findings indicate abundant vascular growth and early tissue reformation in group G.

### 3.7 Systemic Assessment in the Therapeutic Experiment

**FIGURE 6:**
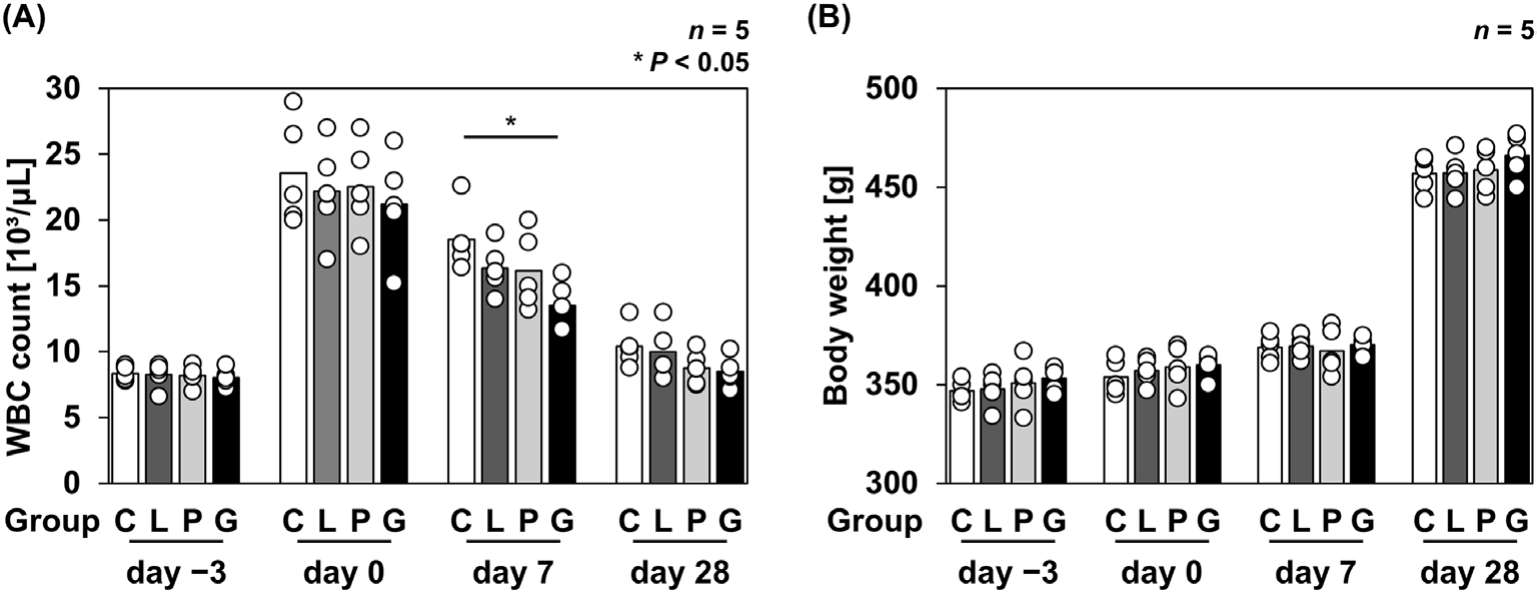
Changes in WBC counts (A) and body weight (B) following four different treatments: Group C, local administration of GM‐PBS; group L, local administration of CEZ solution (250 μg/kg, calculated for CEZ); group P, intraperitoneal injection of CEZ solution (25 mg/kg/day, calculated for CEZ) for 28 days; group G, local administration of GM‐CEZ (250 μg/kg, calculated for CEZ). Bars indicate mean values, and circles indicate individual data points. *p < 0.05; significant difference; Kruskal–Wallis test (*n* = 5). CEZ, cefazolin; GM, gelatin hydrogel microspheres; PBS, phosphate‐buffered saline; WBC, white blood cell.

Blood test results on Day 7 after treatment intervention showed that the WBC count in group G (1.4 ± 0.2 × 10^4^ cells/μL) was significantly lower than that in group C (1.9 ± 0.2 × 10^4^ cells/μL) (*p* = 0.043). The WBC counts in group L (1.6 ± 0.2 × 10^4^ cells/μL) and group P (1.6 ± 0.3 × 10^4^ cells/μL) were relatively smaller than that in group C; however, there were no significant differences (Figure 6A). No significant differences in body weight were observed among the four groups at any time point (Figure 6B). These findings indicate that GM‐CEZ was able to achieve early bacteria suppression without systemic side effects.

### 3.8 In Vivo Systemic Safety Assessments

**FIGURE 7:**
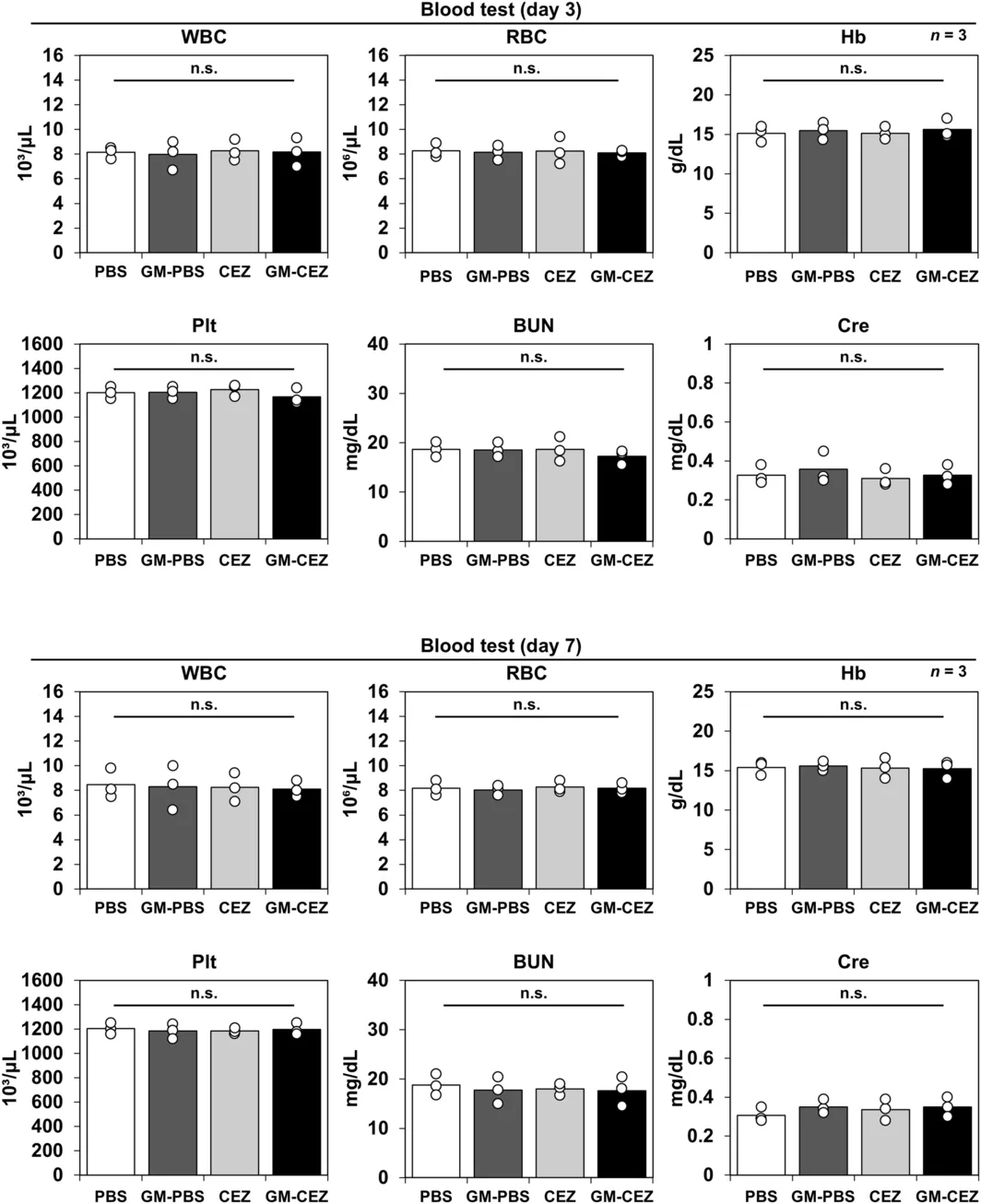
In vivo systemic safety assessment using blood tests. White blood cell count (WBC), red blood cell count (RBC), hemoglobin concentration (Hb), platelet count (Plt), blood urea nitrogen (BUN), and creatinine (Cre) were measured at 3 and 7 days after local injection to the intervertebral disc in the PBS group, GM‐PBS group, CEZ group, and GM‐CEZ group. Bars indicate mean values, and circles indicate individual data points. n.s., not significant; one‐way ANOVA with Tukey–Kramer post hoc test (*n* = 3 per group at each time point). ANOVA, analysis of variance; CEZ, cefazolin; GM, gelatin hydrogel microspheres; PBS, phosphate‐buffered saline.

At both time points, no significant differences in blood tests including hematological and biochemical analyses were observed among PBS‐only group, GM‐PBS group, CEZ group, and GM‐CEZ group (Figure 7). These findings suggest that local administration of GM‐CEZ induced no obvious myelotoxicity or nephrotoxicity.

### 3.9 In Vivo Local Safety Assessments

**FIGURE 8:**
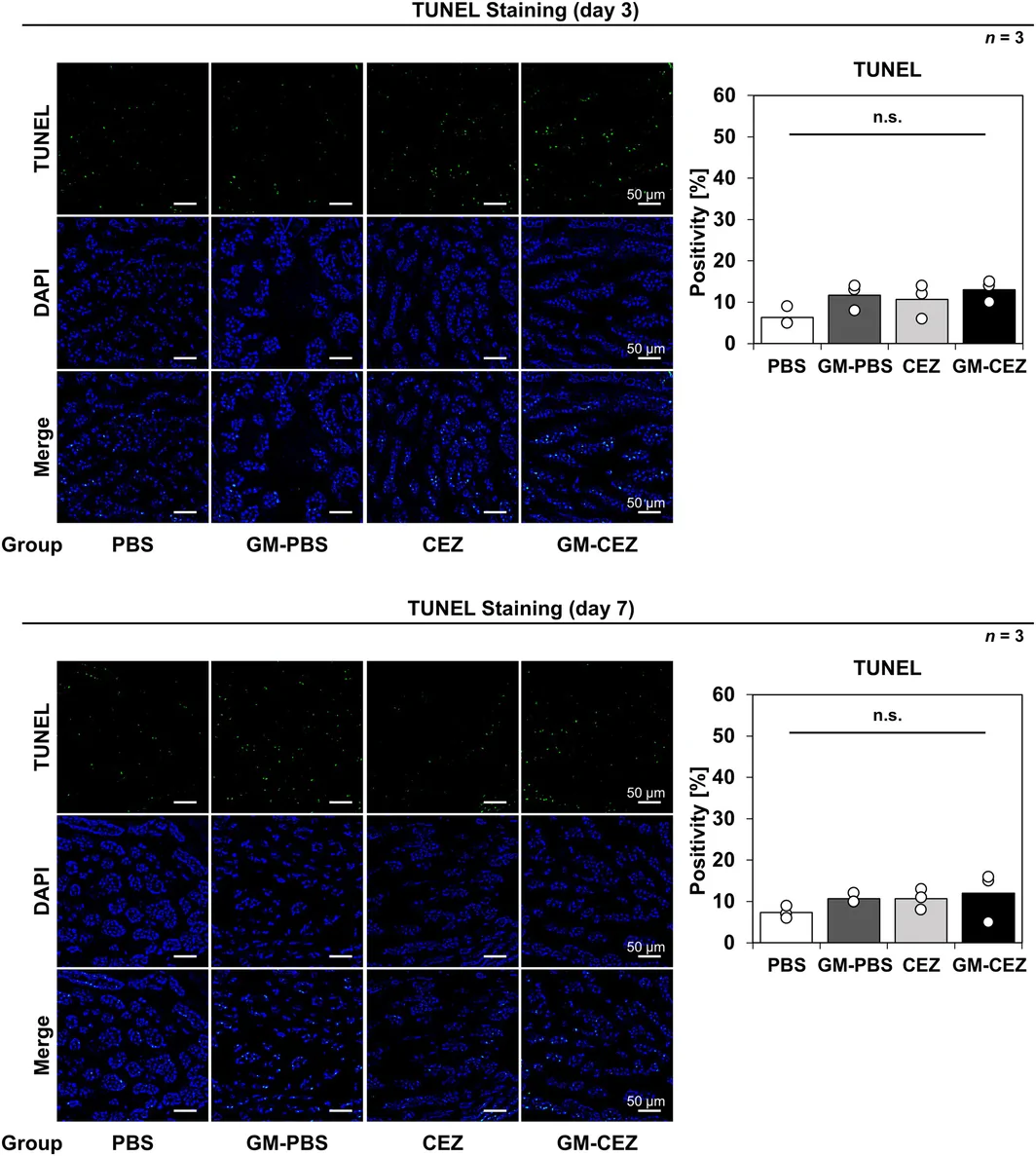
In vivo local safety assessment using TUNEL staining. Representative images of TUNEL staining (green), DAPI nuclear staining (blue), and merged images 3 and 7 days after local injection to the intervertebral disc in the PBS group, GM‐PBS group, CEZ group, and GM‐CEZ group. Quantitative analyses demonstrated no significant differences in the percentage of TUNEL‐positive cells among groups at both time points. Bars indicate mean values, and circles indicate individual data points. n.s., not significant; one‐way ANOVA with Tukey–Kramer post hoc test (*n* = 3 per group at each time point). Scale bar, 50 μm. ANOVA, analysis of variance; CEZ, cefazolin; DAPI, 4′,6‐diamidino‐2‐phenylindole; GM, gelatin hydrogel microspheres; PBS, phosphate‐buffered saline; TUNEL, terminal deoxynucleotidyl transferase dUTP nick end labeling.

At both time points, TUNEL staining demonstrated a low frequency of TUNEL‐positive cells in all groups. At Day 3, the percentages of TUNEL‐positive cells were 6.3% ± 2.3% in the PBS‐only group, 11.7% ± 3.2% in GM‐PBS group, 10.7% ± 4.2% in CEZ group, and 13.0% ± 2.6% in GM‐CEZ group. At Day 7, the percentages were 7.3% ± 1.5%, 10.7% ± 1.2%, 10.7% ± 2.5%, and 12.0% ± 6.1%, respectively. No significant differences were observed among the four groups (Figure 8). These findings suggest that neither the gelatin hydrogel/collagen carrier nor CEZ/GM‐CEZ demonstrated in vivo cytotoxicity in the rat IVD.

## 4 Discussion

This study demonstrated that the local administration of gelatin hydrogel microspheres incorporating cefazolin (GM‐CEZ) enabled sustained release at the infection site, leading to early bacteria suppression, prevention of infection‐induced osteolysis, and early tissue reformation with abundant vascularization without obvious systemic and local toxicity in a rat model of pyogenic spondylodiscitis.

Treating pyogenic spondylodiscitis remains challenging because the avascular nature of the IVD limits antimicrobial drug delivery and therapeutic effect [11, 17]. Moreover, bioactive compounds such as antibiotics have short half‐lives and limited duration of action, further reducing efficacy in this microenvironment [17]. Insufficient antibiotic delivery not only delays recovery but also increases the risk of relapse and antimicrobial resistance [18].

To address these issues, we focused on a topical drug delivery system using gelatin hydrogel microspheres as a sustained‐release carrier [19]. In this study, in vitro analyses confirmed sustained release of the antimicrobial drug from the microspheres while maintaining antimicrobial activity, consistent with previous reports demonstrating preserved bioactivity of therapeutic agents in hydrogel‐based delivery systems [9, 12]. An initial burst release followed by gradual release with gelatin hydrogel degradation also aligns with previous findings using gelatin‐based delivery systems [19]. Furthermore, in vitro experiments using a paper disc soaked in the supernatant from GM‐CEZ demonstrated that the released CEZ retained its bioactivity throughout the degradation process. This result is consistent with prior reports demonstrating the capability of gelatin hydrogel to preserve drug functionality during controlled release [12, 19]. These findings support the feasibility of gelatin hydrogel as a carrier for maintaining therapeutic concentrations of antibiotics at the infection site for a clinically relevant period of time.

Next, we examined the in vivo efficacy of the sustained‐release formulation using a rat spondylodiscitis model. Local administration of GM‐CEZ showed the most effective bacteria suppression in the early phase, as confirmed by IVIS and blood tests. While conventional systemic or free‐form local CEZ treatments also reduced bacterial signals and WBC counts compared to the control, the reduction was relatively less than local administration of GM‐CEZ. Additionally, μCT analysis revealed that infection‐induced bone loss was effectively prevented in rats treated with GM‐CEZ, as evidenced by higher BV/TV values compared to other groups. Early bacteria suppression may lead to preventing osteolytic progression in the infected vertebrae [13]. Taken together, early infection control by local administration of GM‐CEZ may help prevent irreversible structural damage to the spine that may require instrumentation surgery. In addition, immunofluorescence staining for vascular markers, including CD31 and SMA, suggested enhanced vascularization and tissue reformation following GM‐CEZ treatment. The increased vascularization is likely essential for limiting bone erosion and resolving infection, as angiogenesis is tightly linked to tissue reformation [20]. We considered these increases in vascular markers as reflecting enhanced tissue repair within both the vertebral body with osteolysis and the IVD rather than a specific area. Because infection‐induced osteolysis and reparative scar formation rapidly altered the normal anatomical architecture and precise localization of angiogenesis within specific regions was difficult.

Importantly, GM‐CEZ was able to achieve these therapeutic effects using only a fraction of the systemic CEZ dose, thereby reducing the risk of drug‐induced toxicity. Because conventional systemic administration for a long time involves a concern regarding diarrhea and weight loss [21], reduced systemic drug exposure may lower these adverse effects. In fact, no body weight loss was observed in the therapeutic experiments. Moreover, in vivo safety experiments using blood tests including WBC count, RBC count, hemoglobin concentration, platelet count, BUN, and creatinine demonstrated no myelotoxicity and nephrotoxicity [22]. These results suggested systemic safety of GM‐CEZ local administration. Additionally, we also evaluated local safety of GM‐CEZ, because previous studies have suggested that exposure to high local concentrations of antibiotics may impair cellular viability, increase apoptosis, and adversely affect tissue repair [23]. Neither CEZ nor GM‐CEZ local administration into the IVD demonstrated significant increase in apoptotic cells, indicating that local administration of GM‐CEZ exhibited antimicrobial activity without local cytotoxicity in the IVD. Taken together, local administration of GM‐CEZ can minimize the systemic and local side effects while maintaining therapeutic effects.

A few studies have investigated the effectiveness and safety of local drug delivery systems for pyogenic spondylodiscitis. Several approaches, including calcium sulfate beads and PLGA‐based systems, have been explored for local drug delivery in orthopedic and spinal infections [24, 25]. Chitosan‐ and alginate‐based hydrogels have also been investigated as hydrogel‐based drug‐delivery platforms [26, 27]. Compared with these systems, gelatin hydrogel offers several clinically relevant advantages. Alginate‐based hydrogels are commonly formed by ionic crosslinking with divalent cations such as Ca^2+^, and their gel stability and degradation behavior can be influenced by the surrounding ionic environment [26]. Many chitosan‐based formulations require dissolution under mildly acidic conditions before gel formation, which may require careful neutralization to avoid processing‐related biocompatibility concerns [27]. Gelatin hydrogel microspheres used in the present study were dehydrothermally crosslinked without chemical crosslinking agents or ionic gelation or acidic processing, while permitting degradation kinetics to be adjusted through crosslinking conditions such as heating temperature or duration. Compared with PLGA‐based systems, gelatin hydrogel also offers favorable biocompatibility and avoids the use of organic solvents during fabrication. Furthermore, unlike calcium sulfate beads, which typically resorb within 4–8 weeks and may cause sterile wound drainage during dissolution [28], gelatin hydrogel is biocompatible and biodegradable, eliminating the need for surgical removal [29]. The material also allows tunable degradation kinetics and sufficient injectability, making it suitable for minimally invasive and site‐specific applications. Collectively, these characteristics suggest the superior utility of gelatin hydrogel as a platform for local drug delivery in the clinical setting of spinal infections [23, 29]. Previous studies have also demonstrated the utility of gelatin‐based hydrogels in infection control and tissue engineering applications [30, 31]. Furthermore, even if osteolysis has already developed with spinal instability, this novel system may be applicable and can be combined with instrumentation surgery using percutaneous pedicle screws. This system may offer significant clinical and economic benefits, including shorter treatment duration, improved patient's quality of life, and reduced hospitalization [23, 24].

This study has several limitations. First, the findings are based on an acute MSSA infection model in rats; therefore, their applicability to chronic or biofilm‐forming infections, other bacterial species, and optimized protocols for human use remains to be determined [32]. Although CEZ is the standard first‐line treatment for MSSA, further studies using different bacterial species, antibiotics, drug concentrations, administration volumes, and delivery protocols will be required to translate this therapeutic strategy into clinical practice. Our gelatin hydrogel‐based delivery system can be applicable for other antimicrobial agents. Drug release from gelatin hydrogel is governed by both diffusion and matrix degradation, and can be modulated by factors such as crosslinking density. This tunability suggests that the system can be adapted to antibiotics with different molecular sizes and physicochemical properties, including clinically relevant agents such as vancomycin for resistant organisms. However, the release kinetics and stability of each agent may vary. Further optimization will be required for specific drugs. Second, direct mechanical characterization of the present GM‐CEZ/collagen formulation was not performed. Previous studies have reported quantitative mechanical characteristics of gelatin‐containing microspherical materials and gelatin‐based hydrogels. Tris‐acryl gelatin microspheres have been shown to exhibit an approximately linear stress–strain relationship up to 25% deformation, with a Young's modulus of 19.33 ± 4.97 kPa [33]. In addition, gelatin‐based hydrogels have been reported to show viscoelastic and tunable mechanical behavior; neat gelatin gels showed shear storage moduli of approximately 9–13 kPa, and finite‐strain testing demonstrated a tensile tangent modulus of approximately 27 kPa for neat gelatin hydrogels [34]. However, these reported values were obtained from different gelatin‐containing or gelatin‐based hydrogel systems and do not directly represent the mechanical properties of the present GM‐CEZ/collagen formulation. Therefore, we are planning to perform direct rheological, mechanical, and quantitative injectability assessments in future studies. Third, the control design was limited. In the present study, we designed GM‐PBS as the control in the in vivo therapeutic experiments to eliminate the effect of gelatin hydrogel itself, because we focused on the therapeutic effect of controlled‐release CEZ from the gelatin hydrogel delivery system. As a result, our therapeutic experiment lacked a group of local administration of PBS alone. Inclusion of a PBS‐only control group would have provided an additional baseline for comparison, which would strengthen the experimental design.

## 5 Conclusion

In conclusion, local sustained‐release delivery of CEZ using gelatin hydrogel microspheres suppressed infection in the early phase, prevented osteolysis, and promoted tissue reformation without adverse effects in a rat model of pyogenic spondylodiscitis. This strategy may represent a promising approach for local infection control in pyogenic spondylodiscitis, particularly in patients at high risk for surgical intervention and/or prolonged systemic antibiotic therapy.

## Author Contributions

**Kunihiko Miyazaki:** validation, formal analysis, data curation, writing – original draft, writing – review and editing, visualization. **Yoshiki Takeoka:** methodology, investigation, funding acquisition, writing – review and editing. **Masao Ryu:** software, investigation, resources, data curation, writing – review and editing. **Takashi Yurube:** methodology, investigation, funding acquisition, writing – review and editing. **Takeru Tsujimoto:** investigation, resources, data curation, writing – review and editing. **Ryosuke Kuroda:** resources, supervision, project administration, writing – review and editing. **Yutaro Kanda:** conceptualization, validation, writing – original draft, writing – review and editing, visualization, project administration. **Yoshiaki Hiranaka:** software, investigation, resources, data curation, writing – review and editing. **Yasuhiko Tabata:** methodology, supervision, project administration, writing – review and editing. **Hiroki Ohnishi:** software, formal analysis, investigation, resources, data curation, writing – review and editing. **Naotoshi Kumagai:** software, formal analysis, investigation, resources, data curation, writing – review and editing. **Tomoya Matsuo:** software, investigation, resources, data curation, writing – review and editing. **Kohei Kuroshima:** software, formal analysis, investigation, resources, data curation, writing – review and editing. **Kenichiro Kakutani:** conceptualization, resources, supervision, project administration, writing – review and editing.

## Funding

This work was supported by Academic Research Grant of the Hyogo Science and Technology Association (Kobe, Japan), Number 6046.

## Conflicts of Interest

The authors declare no conflicts of interest.

## Data Availability

The data that support the findings of this study are available from the corresponding author upon reasonable request.
